# Nutritional status and its determinants among adolescents with HIV on anti-retroviral treatment in low- and middle-income countries: a systematic review and meta-analysis

**DOI:** 10.1186/s40795-023-00714-z

**Published:** 2023-03-28

**Authors:** Meless Gebrie, Lin Perry, Xiaoyue Xu, Andargachew Kassa, Marilyn Cruickshank

**Affiliations:** 1 College of Medicine and Health Science, Hawassa University, Hawassa, Ethiopia; 2 Faculty of Health, University of Technology Sydney, Ultimo, Australia; 3 Prince of Wales Hospital, Randwick, NSW Australia; 4 School of Population Health, Faculty of Medicine, University of New South Wales, Kensington, NSW Australia; 5 Sydney Children’s Hospitals Network, Sydney, Australia

**Keywords:** Adolescent, Nutrition, Anti-retroviral therapy, Human immunodeficiency virus, Low-middle-income countries, Malnutrition

## Abstract

**Purpose:**

This review aimed to determine what methods are used to assess nutritional status, the levels of nutritional status, determinants of undernutrition, and nutritional interventions employed for adolescents with HIV on Anti-Retroviral Therapy follow-up in Low- and Middle-Income countries.

**Methods:**

Established methods were used to systematically identify and retrieve studies published in five databases between January 2000 to May 2021, and citation searching. Quality was appraised and findings were synthesized using narrative analysis and meta-analysis.

**Result:**

Body Mass Index is the major indicator of nutritional status. The pooled prevalence of stunting, wasting, and overweight were 28.0%, 17.0%, and 5.0%, respectively. Adolescent males are 1.85 and 2.55 times more likely than adolescent females to suffer from both stunting and wasting at AOR = 1.85 (95%:1.47, 2.31) and AOR = 2.55 (95%: 1.88, 3.48), respectively. Similarly, adolescents with a history of opportunistic infections were 2.97 times more likely to be stunted than uninfected adolescents, AOR = 2.97 (95%:1.73, 5.12). One single intervention study found significant improvements in anthropometric status after nutritional supplementation.

**Conclusion and recommendation:**

The few studies that have been conducted on nutritional status in adolescents living with HIV in low- and middle-income countries indicate that stunting and wasting are common in this population. Avoiding opportunistic infections is an important protective factor but the review highlighted the generally inadequate and fragmented nature of nutritional screening and support programs. Development of comprehensive and integrated systems for nutritional assessment and intervention services during ART follow-up should be prioritized to improve adolescent clinical outcomes and survival.

**Supplementary Information:**

The online version contains supplementary material available at 10.1186/s40795-023-00714-z.

## Introduction

Adolescence (ages 10 to 19 years [1]) is a critical time period since many of the risk factors for adult diseases develop during this age [2]. This age group provides a second window of opportunity for positive life cycle development and aids in mitigating adult problems [3]. Adolescents account for 16% of the global population but comprise a quarter of the population in some countries. Numbers are expected to rise through 2050, particularly in low- and middle-income countries (LMICs). More than half of all adolescents live in Asia but in sub-Saharan Africa they make up the greatest proportion of the population, at 23% [1, 4].

Adolescents are disproportionately affected by the human immunodeficiency virus (HIV) [1, 3]. In 2020, about 1.75 million adolescents worldwide were living with HIV, representing approximately 5% of all people with HIV, 11% of new HIV infections, and 5% of all AIDS-related deaths. In the same year, approximately 940,000 adolescents, 54% of adolescents living with HIV worldwide, received antiretroviral therapy (ART) [3, 4]. More than 1.5 million adolescents and young adults aged 10 -24 years die each year, nearly 5000 every day, from largely preventable causes; three-fourths occur in LMICs [5]. HIV is a preventable cause of disability, morbidity, and mortality among adolescents and young people, with an increasing proportion reporting depression, stigma, violence, and suicidal behaviour [3–5].

Adolescents living with HIV are vulnerable to undernutrition due to their elevated nutritional needs imposed by a puberty growth spurt and HIV infection. Malnutrition is a major threat to the health of HIV-infected individuals and is associated with increased risks of morbidity and mortality [6]. Despite the introduction by the United Nations of Sustainable Development Goals (SDG), designed to safeguard the most vulnerable [7], and the decreasing trends seen in many communicable and nutritional disorders, malnutrition remains a major public health concern [2]. Moreover, progress has been inequitable with countries with a low and low–middle social development index (SDI) bearing a higher burden of morbidity amongst children and adolescents compared to middle-, high–middle-, and high-SDI countries [2]. About 88% (1.5 million) of all HIV-infected adolescents live in sub-Saharan Africa and although health-related initiatives have been instigated through SDG-aligned legislation, these adolescents still face severe health vulnerabilities [8].

In many LMICs, the targets for adolescents’ physical health predominantly focuses on the sexual and reproductive health (SRH) behaviors of young people aged over 15. However, nutrition plays a critical role not just for SRH but also in the life cycle, transitioning from adolescence to healthy adults. Malnutrition among children and adolescents is associated with delayed growth, impaired cognitive maturation, lower intellectual quotient, behavioral problems and increased risk of contracting communicable disease [1, 9, 10]. Further, younger adolescents with HIV have greater nutritional and health demands because they face extra challenges beyond those caused by the general health vulnerabilities affecting adolescents in LMICs, such as cultural/norm-based practices, gender-based violence, and heavy workloads [11]. Due to their increased nutritional requirements, those living with HIV are particularly susceptible to undernutrition. Adequate and proper nutrition for well-nourished patients with HIV leads to slower disease progression than experienced by those who are malnourished but teenagers are rarely given priority in nutrition initiatives and there is little research on the epidemiology of undernutrition and its causes among adolescents living with HIV in LMICs. This review, therefore, aimed to determine how nutritional status is assessed and to evaluate the nutritional status of HIV-positive adolescents and its determinant factors in LMICs.

## Methods and materials

### Study design

An integrative review design was used as it was anticipated that evidence would derive from a variety of quantitative and qualitative studies.

The Preferred Reporting Items for Systematic Reviews and Meta-Analysis (PRISMA) guideline was followed to report results [12] (see Supplementary Table 1).

### Research questions

1. What methods are used to assess nutritional status of adolescents who are HIV positive on ART follow-up living in LMICs?
2. What is the nutritional status of adolescents who are HIV positive on ART follow-up living in LMICs?
3. What are the determinant factors associated with the nutritional status of adolescents who are HIV positive and on ART follow-up living in LMICs?
4. What nutritional interventions, if any, have a significant improvement on the nutritional status of adolescents who are HIV positive and on ART follow-up living in LMICs?

### Search strategy and sources of information

The “Population, Intervention, Comparator, Outcomes (PICO)” and “Population, Exposure, Outcomes (PEO)” frameworks were used to develop robust literature search strategies. After a preliminary assessment of the appropriate Medical Subject Heading terms (Title-Abstract-Keywords), keywords and synonyms, a search strategy was developed. As the terms nutrition screening and assessment were often used interchangeably in studies, synonyms of *malnutrition* and *nutritional status* were combined with synonyms of *screening* and *assessment *(see Supplementary Table 1).

The PICO/PEO framework was applied for research question 1 & 2 as follows:

◦ In adolescents who are HIV positive and on ART follow-up living in LMICs (P), what methods of assessment were used (I) to determine the level of nutritional status (O) compared with WHO standards (C)?
◦ In adolescents who are HIV positive on ART follow-up living in LMICs (P), what levels of nutritional status (O) were reported compared with WHO standards (C)?

To address research question 3:

◦ In adolescents who are HIV positive and on ART follow-up living in LMICs (P), what determinant factors (I or E) lead to a changed (increased/decreased) risk of malnutrition/undernutrition (O)?

To address research question 4 effectiveness studies were sought to determine:

◦ What nutritional interventions (I) affect the nutritional status outcomes (O) of adolescents who are HIV positive on ART follow-up living in LMICs (P) compared to comparison group (C) outcomes?

The search strategy was applied, with individual modifications, to the electronic databases: Medline (Ovid), PubMed, ProQuest, EMBASE (Ovid), and Cochrane Library of Databases. Records were systematically searched for publications from January 2000 to May 2021 for materials that met the inclusion criteria. In addition, citations/reference lists of retrieved relevant articles were searched. Additional articles were advance-searched from Web of Science and Google Scholar.

#### Inclusion criteria

##### Study setting

Studies conducted in LMICs, classified according to the World Bank criteria [13, 14]

##### Population

The target population was adolescents who were HIV positive and on ART follow-up. For this review, participants were required to be specified as:

- Aged between 10—19 years of age, or
- If age was not specified, participants were referred to as older children OR young adults OR teenagers OR young person’s OR young people whose age was 10 – 24 years, or
- If the sample was of mixed age (children/ adolescent/ young adult) the mean or median age or most of the sample (> 50%) must lie between 10–19 years

##### Type of publication

Primary studies, both published and grey literature in the English language

##### Study design

To address the first three questions, prospective studies, retrospective studies, cross-sectional studies, descriptive/quantitative and experimental studies, qualitative studies, and mixed-methods studies were sought

To address the fourth question effectiveness designs, such as experimental studies, randomized controlled trials (RCT), controlled clinical trials, quasi-experimental studies, or other interventional study designs were required.

#### Exclusion criteria

- Conference abstracts and other studies which did not have the full text available
- Reviews and other forms of report using secondary analysis.

### Study screening, selection, and data extraction

**Fig. 1:**
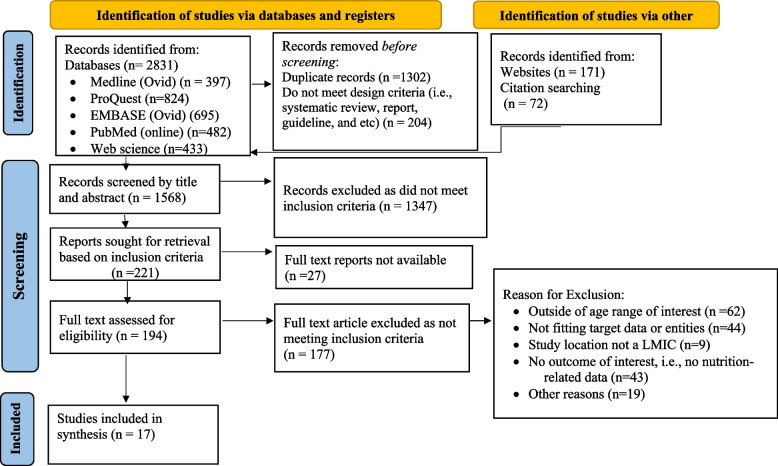
Preferred Reporting Items for Systematic Reviews and Meta-Analyses (PRISMA) flow diagram of the review [12] (see Supplementary Tables 2 and 3)

The results of the search were exported to an Endnote library Version 20 after which duplicate items were automatically eliminated. Article titles, abstracts, and keywords were reviewed by two independent reviewers (MG & AK) for evaluation against the inclusion and exclusion criteria for eligibility. The complete texts of all potentially relevant papers were retrieved, and their citations were uploaded to the Joanna Briggs Institute System for the Unified Management, Assessment, and Review of Information (JBI SUMARI) for full-text screening (JBI, Adelaide, Australia). For articles that did not have full details, authors were contacted to obtain the full-text report at the corresponding author addresses. Papers were retained for full-text evaluation in cases where eligibility was ambiguous. Studies that were ineligible were excluded, with reasons noted. At each level of the selection process, any discrepancies between the reviewers were settled through conversation or by consulting a third reviewer (MC, LP). The outcomes of the search and screening procedure were displayed in a PRISMA flow diagram (Fig. 1).

Data were extracted by MG, AK, and AC/MC, and verified by LP and XX. Two reviewers extracted data for the quantitative component from the quantitative and mixed methods (quantitative component only) studies related to study contexts and participants, research techniques, interventions, and outcomes relevant to review questions. Data were taken verbatim where possible and any discrepancies between the reviewers were settled through conversation or by consulting a third reviewer. When necessary, data were requested from the study authors.

### Quality appraisal

The selected studies were critically evaluated by two independent reviewers for methodological quality using the JBI appraisal instruments [15, 16]. Reviewers used the appropriate JBI quality assessment tool for each individual study design. Any disagreements between reviewers were resolved through discussions or with a third reviewer (see Supplementary Table 4).

### Data synthesis and analysis

Extracted data were exported from Microsoft Excel to STATA Version 17.0 (software) for analysis. The characteristics of included studies and descriptive results were presented using tables and graphs. A random effects meta-analysis model was used and the pooled effect size was employed. Forest plots were used to show the pooled estimates with 95% confidence intervals (CI). The strategy for meta-analysis was based on the guidance of the Campbell systematic review model [17]. Publication bias and heterogeneity of the studies was assessed. Publication bias was assessed by examining a funnel plot of the data and performing Egger’s test [18] to determine whether the effect size correlated with their standard errors. A sign of publication bias was considered in this study when studies with larger standard errors lead to larger effect sizes. Sub-group analysis was conducted to examine data heterogeneity and Cochran’s Q-test and I2 statistics were calculated to estimate the level of heterogeneity.

## Results

After duplicates were removed, a total of 1568 articles remained for Title-Abstract screening. Of these, 1347 articles were excluded as they did not meet inclusion criteria, and 221 articles remained for full-text screening. Of these 221 articles, 27 were removed because the full report was not available and 177 were excluded for not meeting the inclusion criteria. In total, 17 articles were retained for data extraction and synthesis (Fig. 1).

### Characteristics of included studies

These 17 primary articles recruited a total of 2873 study participants in LMICs. The majority of articles (*n* = 11, 64.7%) were cross-sectional studies [19–27], whilst the remainder (*n* = 6, 35.3%) comprised: observational studies (*n* = 3) [28–30]; case–control study (*n* = 1) [31]); mixed-method study (*n* = 1) [32], and one clinical trial [33] (Table 1).

**Table 1:** Summary of study characteristics (*n* = 17)

**Variable with Category**	**Number of studies**	**Percentage (%)**
**Study setting by region**		
African countries *[Cambodia (n* = *1), Cameroon (n* = *1), central and west Africa (n* = *1), Ethiopia (n* = *2), Senegal(n* = *1), Uganda(n* = *2)]*	8	47.1
Asian countries *[India (n* = *1) and Myanmar (n* = *1)]*	2	11.8
South America *[Brazil (n* = *7)]*	7	41.2
**Country Income**		
Low-Income country	5	29.4
Low-Middle Income Country	5	29.4
Upper-Middle Income Country	7	41.2
**Study Design**		
Case Control Studies	1	5.9
Clinical trial based interventional study	1	5.9
Cross-sectional study	11	64.7
Mixed Method study	1	5.9
Observational Study	3	17.6
**Sample size**		
< 100 participants	7	41.2
≥ 100—200 participants	5	29.4
> 200 Participants	5	29.4

Studies were conducted in seven LMIC countries in South American, Asian and African continents. The majority (*n* = 7, 41.2%) took place in upper-middle income countries [19, 21, 22, 27, 28, 30, 34], followed by low-middle income countries (*n* = 5, 29.4%) [26, 29, 32, 33] and low- income countries (29.4%) [20, 23–25, 35] (Table 1).

### Outcome 1, review question 1: nutritional assessments

Of the 17 included studies, the majority (*n* = 13, 76.5%) used BMI as an indicator of nutritional status, followed by height for age (*n* = 7, 41.2%). Six (35.3%) studies assessed dietary intakes and estimated energy and nutrient intakes, and three studies (17.6%) assessed body fat composition using standardized fat measurement (Table 2).

**Table 2:** Methods of nutritional assessment used in included studies (*n* = 17)

**Indices**	**Types of Nutritional Assessment**	**Studies (n)**	**Percentage (%)**
**Anthropometric indices**	Height-for-age (HAZ)	7	41.2
	Body mass index (BMI) Z-score	13	76.5
	Weight-for-height Z-score	1	5.9
	Weight-for-Age Z-score	1	5.9
**Body composition / Body Fat assessment**	Skinfolds thickness (abdominal, triceps, subscapular, calf)	3	17.6
	Waist-to-height ratio (WHR)	1	5.9
	Waist and hip circumferences (WHC)	3	17.6
	Perimeter relaxed arm (PRA)	1	5.9
	Perimeter neck / Neck circumference-for-age	2	11.8
	Air displacement plethysmography (ADP)	1	5.9
	Body adiposity index (BAI)	1	5.9
	Dual-energy X-Ray absorptiometry (DEXA)	1	5.9
	Conicity index	1	5.9
	Lipodystrophy physical diagnosis for abnormal fat distribution	1	5.9
	Bone Mineral Density (BMD)/ Bone mineral content (BMC)	2	11.8
	Body fat percentage	3	17.6
	Lean mass	1	5.9
	Upper-arm fat area	1	5.9
	Upper-arm muscle area	1	5.9
**Dietary assessment**	Food frequency questionnaires (FFQ) for dietary assessment to estimate total energy intake (TEI) and nutrient intake	1	5.9
	24-h recall Dietary Intake Assessment to estimate Energy and nutrient intake	4	23.5
	Individual Dietary Diversity status	1	5.9

### Outcome 2, review question 2: nutritional status of study participants

The majority of studies (*n* = 10, 58.8% and *n* = 9, 52.9%, respectively) reported nutritional status in terms of stunting and wasting; six studies (*n* = 6, 35.3%) reported on overweight status. In most studies BMI-for-age Z-score and Height-for-Age Z-score (HAZ) below -2 Z score were used to determine nutritional status (under-weight, wasting, stunting and overweight) but a few articles used Weight-for-Age Z-score (WAZ) and Weight-for-Height Z-scores (WHZ) below − 2 Z- scores (WHO standard).

#### Prevalence of stunting

Most studies that reported stunting demonstrated that it occurred in between one fifth and one third of the participants (*n* = 7 of 9, ranging 20.9 – 36.6%). The highest prevalence of stunting, at 46.6%, was reported from a study in Cambodia [26] and the lowest, at 6.1%, was from Brazil [30] (Table 3).

**Table 3:** The prevalence of stunting among adolescents living with HIV and on ART follow-up in LMICs (*n* = 9)

**Author Name**	**Year**	**Country**	**Sample size**	**Stunting** **n (%)**
Darshit, et al. [20]	2020	Uganda	132	31(23.7)
David, et al [31]	2020	Cameroon	75	15(36.6)
Dos Reis, et al [21]	2015	Brazil	115	24(20.9)
Hillesheim, et al [30]	2014	Brazil	49	3(6.1)
Jesson, et al [23]	2015	Central and West-African	684	163(23.8)
Lwanga, et al [25]	2015	Uganda	200	72(36.2)
Ramalho, et al [34]	2011	Brazil	94	24(25.5)
Shiferaw & Gebremedhin [24]	2020	Ethiopia	260	86(33.1)
Yasuoka, et al [26]	2020	Cambodia	298	139(46.6)

#### Prevalence of wasting

More than half the studies reported wasting in between one tenth and one fourth of the samples (*n* = 5 of 9, ranging 10.0 – 22.3%). The highest prevalence of wasting was reported in a study conducted in Ethiopia (60.2%) [35], and the lowest was in Brazil (2.2%) [30] (Table 4).

**Table 4:** The prevalence of wasting among adolescents living with HIV and on ART follow-up in LMICs (*n* = 9)

**Author Name**	**Year**	**Country**	**Sample size**	**Wasting** **n (%)**
Darshit, et al [20]	2020	Uganda	132	10(7.6)
Dos Reis, et al [21]	2015	Brazil	115	4(3.5)
Hillesheim, et al [30]	2014	Brazil	49	1(2.0)
Jesson, et al [23]	2015	Central and West-African	684	69(10.0)
Lwanga, et al [25]	2015	Uganda	200	36(18.0)
Ramalho, et al [34]	2011	Brazil	94	21(22.3)
Sewale, et al., [35]	2018	Ethiopia	372	224(60.2)
Shiferaw & Gebremedhin [24]	2020	Ethiopia	260	52(20.0)
Yasuoka, et al [26]	2020	Cambodia	298	39(13.1)

#### Prevalence of overweight

Most of the studies demonstrated that overweight was the least common findings of nutritional assessment (*n* = 4 of 6, ranging from 6.1%—15.6%). The highest (15.6%) and lowest (1.9%) prevalence of overweight were reported from studies conducted in Brazil [21] and Ethiopia [24], respectively (Table 5).

**Table 5:** The prevalence of overweight among adolescents living with HIV and on ART follow-up in LMICs (*n* = 6)

**Author Name**	**Year**	**Country**	**Sample size**	**Overweight n (%)**
Darshit, et al [20]	2020	Uganda	132	4(3.8)
David Chelo, et al [31]	2020	Cameroon	75	3(7.3)
Dos Reis, et al [21]	2015	Brazil	115	18(15.6)
Hillesheim, et al [30]	2014	Brazil	49	3(6.1)
Ramalho, et al [34]	2011	Brazil	94	6(6.4)
Shiferaw and Gebremedhin [24]	2020	Ethiopia	260	5(1.9)

### Outcome 3, review question 3: factors associated with undernutrition among adolescents living with HIV

Variables reported as significantly associated with stunting and wasting in at least two primary studies were included in this meta-analysis. Accordingly, being of male sex and having opportunistic infection were found to be significantly associated with stunting [23–25, 35]. Only male sex was a significant factor for wasting /thinness [23, 26, 35] (Table 6).

**Table 6:** Factors associated with undernutrition among adolescents living with HIV in LMICs

Types of Undernutrition	Variable	Number of studies	Studies included in the analysis	Pooled Odds Ratio with 95%CI	Heterogeneity	
					(I^2^)	*P*-Value
Stunting	Sex (male Sex)	3	[23]	1.847(1.474, 2.313)	72.60%	*P* = 0.026
			[25]			
			[35]			
	Opportunistic infection	2	[35]	2.97(1.73, 5.12)	31.90%	*p* = 0.225
			[24]			
Wasting / thinness	Sex (Male Sex)	3	[23]	2.55(1.88, 3.48)	34.90%	*p* = 0.215
			[35]			
			[26]			
	Opportunistic infection	2	[35]	3.70(2.12, 6.45)	0.00%	*p* = 0.569
			[24]			

A total of 1261 participants were included to analyze the association between sex and stunting among adolescents living with HIV in LMIC. The pooled odds ratio showed that male adolescents were 1.847 times at greater odds of stunting than their female counterparts (AOR = 1.847 (95%CI: 1.474, 2.313), I^2^ = 72.6%, *P* = 0.026) [23, 25, 35]. Six hundred and thirty-two participants were included to analyze the association between a history of opportunistic infection and stunting, demonstrating adolescents who had an opportunistic infection were 2.97 times more likely to develop stunting than their non-infected counterparts (AOR = 2.97 (95%CI: 1.73, 5.12), I^2^ = 31.9%, *P* = 0.225) [24, 35] (Table 6).

A total of 1354 participants were included to analyze the association between sex and wasting among adolescents living with HIV in LMIC. The pooled odds ratio showed that male adolescents were 2.55 times more likely to become wasted / thin compared to their female counterparts (AOR = 2.55(95%CI: 1.88, 3.48), I^2^ = 34.9%, *P* = 0.215) [23, 26, 35] (Table 6).

### Outcome 4, review question 4: nutritional interventions, outcomes and the magnitude of effect

Only one interventional study was found, trialling nutritional supplementation of 360 kcal energy and 32.2gm protein in food made from Peanut Chikki and another source. The study reported that after one year of supplementation on a daily basis the study participants showed a significant improvement in their Height-for-Age, Weight-for-Age and BMI-for-Age indices [29].

### Meta-analysis

**Fig. 2:**
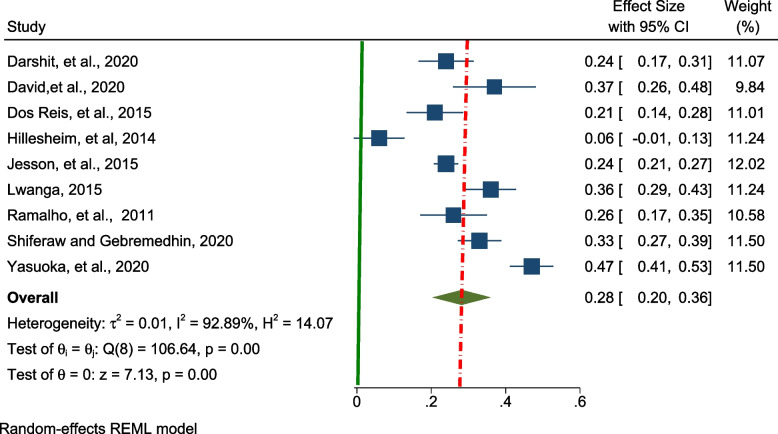
Forest plot for the pooled prevalence of stunting among adolescents living with HIV in LMICs (*n* = 9)

A random effect meta-analysis model was used to estimate the pooled prevalence of under-nutrition among adolescents living with HIV in LMIC. To estimate the prevalence of stunting, nine studies were included in the analysis and the overall pooled prevalence of stunting was 28.0% (95% CI; 20.0–36.00, I^2^ = 92.89%, *p* < 0.01), (Fig. 2).

**Fig. 3:**
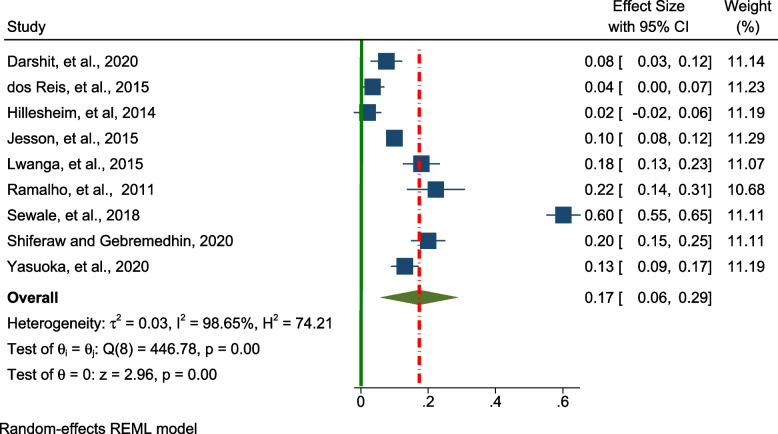
Forest plot for the pooled prevalence of wasting among adolescents living with HIV in LMICs (*n* = 9)

Similarly, nine studies were included in the analysis to estimate the pooled prevalence of wasting /thinness, which was demonstrated as 17.0% (95% CI; 0.06– 0.29, I^2^ = 98.65% *p* < 0.01), (Fig. 3).

**Fig. 4:**
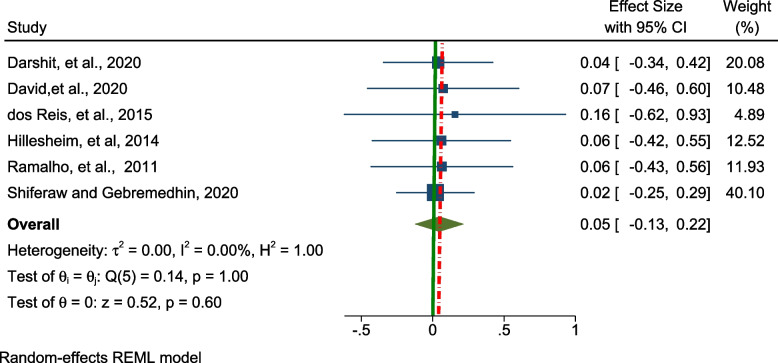
Forest plot for the pooled prevalence of overweight among adolescents living with HIV in LMICs (*n* = 6)

Regarding overweight, a fixed effect model was used to estimate the pooled prevalence of overweight. Six studies were included in this analysis and the overall pooled prevalence of overweight amongst the adolescents was 5.0% (95% CI; -0.13 – 0.22, I^2^ = 0.00, *p* = 0.6), (Fig. 4).

### Sub-group meta-analysis and heterogeneity summary

Subgroup analysis was performed using country and study design to identify the source of heterogeneity.

#### Sub-group meta-analysis and heterogeneity summary for stunting

Subgroup analyses were conducted by country for Brazil [21, 30, 34] and Uganda [20, 25]. From these studies, the highest pooled prevalence estimate of stunting was in Uganda, at 30.1% (95% CI: 18.3–41.8, I^2^ = 82.47%, *P* < 0.001) and the lowest was in Brazil, at 17.40% (95% CI: 5.50–29.30, I^2^ = 86.35, *P* = 0.004).

Sub-group analysis was also conducted by study design for cross-sectional studies, and the pooled prevalence estimates of stunting by this design was 30.03% (95% CI: 23.4–37.10, I^2^ = 89.58, *P* < 0.001) [20, 21, 23–26, 34] (Table 7).

**Table 7:** Subgroup meta-analysis for the percentage of prevalence effect size of stunting among adolescents living with HIV in LMICs

Sub-group by category type	Studies (n)	Pooled prevalence % ES (95%CI) with Test of Differences within each subgroup					
		% ES (95% CI)	*P*-Value	Q	I^2^	Tau^2^	H^2^
Sub-group by country							
Brazil	3	17.4 (5.5, 29.3)	0.004	15.32	86.35	0.009	7.33
Cambodia^a^	1	47.0 (41.0, 53.0)	< 0.001				
Cameroon^a^	1	37.0 (26.0, 48.0)	< 0.001				
Central and West-African^a^	1	24.0 (21.0, 27.0)	< 0.001				
Ethiopia^a^	1	33.0 (27.0, 39.0)	< 0.001				
Uganda	2	30.1 (18.3, 41.8)	< 0.001	5.7	82.47	0.006	7.30
Sub-Group by study design							
Case control study^a^	1	37.0 (26.0, 48.0)	< 0.001			0.000	
Cross-sectional study	7	30.3 (23.4, 37.1)	< 0.001	61.09	89.58	0.008	9.60
Observational study^a^	1	6.0 (-7.0, 12.7)	0.078			0.000	
Study Population							
Only Adolescent age population	4	32(25.0, 38.0)	< 0.001	17.34	78.47	0.00	4.64
Mixed age population	5	25(12.0, 38.0)	< 0.001	88.87	94.22	0.02	17.3

*CI* Confidence Intervals, *ES* Effect Size

^a^Countries and study designs having single study

Furthermore, sub-group analysis was conducted by study population, and the pooled prevalence estimate of stunting for only adolescent age study population was 32.0% (95%CI:25.0, 38.0; I^2^ = 78.47, *p* < 0.001) [23–25, 31], whereas the pooled prevalence estimate of stunting for mixed age study population was 25.0% (95%CI:12.0, 38.0; I^2^ = 88.87, *P* < 0.001) [20, 21, 26, 30, 34]. Regarding the test of difference within each subgroup analysis, there significant heterogeneity within each sub-group based on the statistical significance of I^2^ statistics, the Cochran’s’Q’ result and *p*-values, as indicated in Table 7 below.

#### Sub-group meta-analysis and heterogeneity summary for wasting

Subgroup analyses were conducted by country for Brazil [21, 30, 34], Ethiopia [24, 35] and Uganda [20, 25]. From these studies, only the pooled estimate for Uganda provided evidence of significant heterogeneity, at 12.7% ((95% CI: 2.5–22.9), I^2^ = 88.37%, *P* < 0.001).

Sub-group analysis was also conducted by study design for cross-sectional studies, and the pooled prevalence estimates of wasting by this design was 19.3% ((95% CI: 7.0–31.60), I^2^ = 98.66, *P* < 0.01) [20, 21, 23–26, 34, 35] (Table 8).

**Table 8:** Subgroup meta-analysis for the percentage of prevalence effect size of wasting among adolescents living with HIV in LMICs

**Sub-group by category type**	**Studies (n)**	**Pooled prevalence % ES (95%CI) with Test of Differences within each subgroup**					
		**%ES (95% CI)**	***P*-Value**	**Q**	**I**^**2**^	**Tau**^**2**^	**H**^**2**^
**Sub-group by country**							
**Brazil**	**3**	8.7 (-3.5, 21.0)	0.162	19.04	95.22	0.011	20.91
**Cambodia**^a^	**1**	13.1 (9.2, 17.0)	< 0.001				
**Central and West-African**^a^	**1**	10.0 (7.8, 12.2)	< 0.001				
**Ethiopia**^a^	**2**	40.1 (0.7, 79.5)	0.045	129.28	99.23	0.08	129.28
**Uganda**	**2**	12.7 (2.5, 22.9)	0.015	8.6	88.37	0.005	8.6
**Sub-Group by study design**							
**Cross-sectional study**	**8**	19.3(7.0, 31.6)	0.002	412.0	98.66	0.031	74.41
**Observational study**^a^	**1**	2.0 (-1.9, 5.9)	0.317			0.000	
**Study population**							
**Only adolescent age population**	**3**	15.6(9.3, 22.0)	0.000	18.3	86.34	0.003	7.32
**Mixed Age Population**	**6**	18.1(1.0, 35.6)	0.000	427.29	98.96	0.047	96.14

*CI* Confidence Interval, *ES* Effect Size

^a^Countries and study designs having single study

Furthermore, sub-group analysis was conducted by study population, and the pooled prevalence estimate of wasting for only adolescent age study population was 15.6% (95%CI:9.3, 22.0, I^2^ = 86.34, *p* < 0.0001) [23–25], whereas the pooled prevalence estimate of wasting for mixed age study population was 18.1% (95%CI:1.0, 35.6, I^2^ = 98.96, *P* < 0.0001) [20, 21, 26, 30, 34, 35].

Regarding the tests of differences within each subgroup analysis, there was evidence of significant heterogeneity observed within each sub-group based on the statistical significance of I^2^ statistics, Cochran’s’Q’ results and the *p*-values indicated in Table 8 below, but no significant difference was observed on pooled estimates by country for Brazil and Ethiopia.

### Quality of included studies

The average JBI quality score of included studies was 6.76 (95% confidence interval 6.2- 7.5); the minimum quality score was 5 and maximum was 10 (the maximum possible quality score). A score of 7 and above was described as indicative of good quality, and 10 (58.8%) of the articles scored at this level (see Supplementary Table 4).

**Fig. 5:**
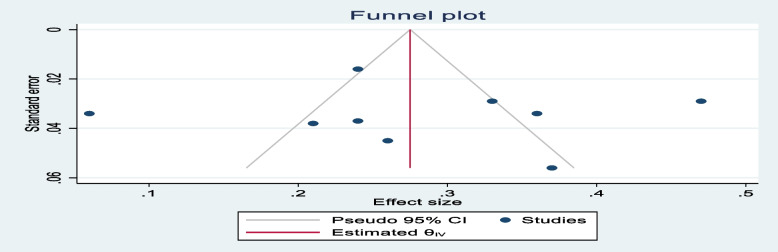
Funnel plot showing the symmetric distribution of articles analyzed for pooled prevalence of stunting among adolescents living with HIV in LMICs

**Fig. 6:**
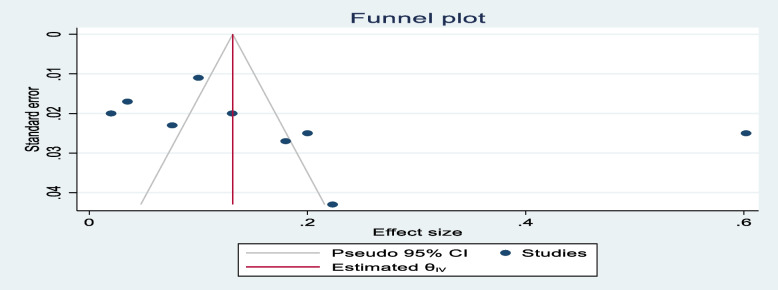
Funnel plot showing the symmetric distribution of articles analyzed for pooled prevalence of wasting among adolescents living with HIV in LMICs

**Fig. 7:**
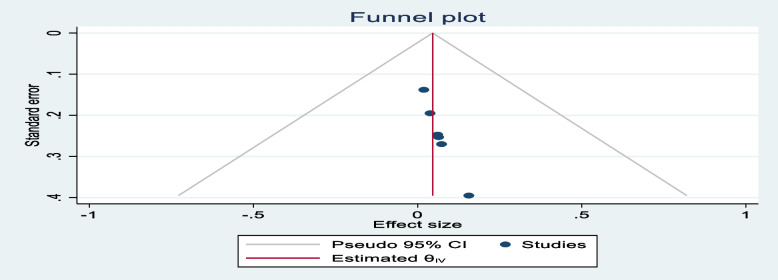
Funnel plot showing the symmetric distribution of articles analyzed for pooled prevalence of overweight among adolescents living with HIV in LMICs

Publication bias was checked using Egger’s test and the results showed no significant publication bias, as evidenced by *p* = 0.865, 0.055, and 0.735 for stunting, wasting, and overweight, respectively. The symmetrical distribution of the funnel plots indicated that publication bias was not a significant problem in this meta-analysis (Figs. 5, 6, and 7).

## Discussion

This review shows that evidence of undernutrition among adolescents living with HIV in LMICs is scarce. This is an important omission because most such adolescents are at risk of undernutrition secondary to an elevated nutritional need imposed by their age-related growth spurt and HIV infection. Further, undernutrition may predict disease progression in HIV-infected individuals and result in a higher risk of morbidity and mortality in both HIV-infected adolescents and adults. Consequently, efforts to treat HIV infection are less likely to achieve good outcomes for the individual (and their community) if underlying malnutrition is not also addressed [36]. Whilst no study examined this, the cost-effectiveness of the treatment of HIV may be severely impacted in the absence of treatment of underpinning vulnerability factors such as malnutrition.

This review revealed the narrow variety of nutritional assessment techniques in use with adolescents with HIV on ART. Most studies used anthropometric assessments, most commonly BMI-for-Age (76.5%) and Height-for-Age (41.2%). These anthropometric tools are inexpensive, portable, simple to use, and require minimal training, but are less sensitive and specific indicators of nutritional status. A few studies (17.6%) also conducted body fat composition measurements, using various standardized tools, such as skin-fold thickness, circumference measurement (Waist/hip circumference), Bioelectrical Impendence Analysis (BIA), Dual-energy X-ray Absorptiometry and Air Displacement Plethysmography (ADP) [21, 22, 27]. These measurement tools have better ability to determine nutritional status and to differentiate fat from fat-free mass but are used much less often in LMICs, based on the review findings. One possible reason for this might be the scarcity of such resources and personnel trained to use them.

Some studies (35.3%) evaluated dietary intake, using one-time 24-h dietary review (*n* = 4), FFQ (*n* = 1) and individual assessment of dietary diversity (*n* = 1) to identify energy and nutrient intake. There are significant challenges in obtaining accurate diet histories as the tools are arduous to complete and of little value unless there is a high degree of completeness and accuracy. Use of such limited methods in the determination of nutritional status results in limited information and difficulty in differentiating whether apparent undernutrition was a result of disease effects, inadequate food intake or other predisposing factors.

This review revealed a high pooled prevalence of stunting and wasting and a low prevalence of overweight in this population. Among the seventeen studies included in this meta-analysis, nine reported the prevalence of stunting with the range from 6.1% to 46.6%. This significant discrepancy in the studies may be caused by a varied but generally limited emphasis on nutrition treatment and support for undernourished children and adolescents, lack of standardized and integrated service provision for HIV-positive individuals and poor screening in clinical and nutrition-related conditions during ART follow-up at health facilities [37]. Further the different research methods used in these studies may have contributed to variability in findings. The overall pooled prevalence of stunting in this systematic review meta-analysis was 28.0% among adolescents living with HIV in LMIC. This finding was lower than that seen in the large-scale study conducted among HIV infected adolescents (41%) in sub-Saharan Africa, the Asia–Pacific, Caribbean, Central and South America regions of the world [38]. Discrepancies may result from differences in samples and sample sizes, study design and settings, and socio-cultural differences (e.g. socio-economic status, dietary habits and health services) between studies.

Nine of these seventeen studies reported the prevalence of wasting, ranging from 2% to 60.2%. In many LMICs, including Ethiopia with the highest reported prevalence of wasting, integrated HIV care and support services are recent developments for adolescents living with HIV during ART follow-up. Weak policy implementation, resulting in inadequate provision of nutrition screening, counselling and supplementation, may all contribute to the high rates of wasting seen amongst this population in many LMICs. Discrepancies between studies and countries might be related to differences in sociocultural and/ or socioeconomic characteristics, health care providers’ knowledge, attitudes and skills, health facilities and health systems, and study methods and settings. However, the pooled prevalence of wasting, 12.0% in this meta-analysis, is similar to that in another study from less developed regions of the world (14.5%) [38].

Six of these seventeen studies revealed the prevalence of overweight, with the pooled prevalence of 5.0% and the highest and lowest frequencies reversing the findings for wasting. A study in Brazil found the highest frequency of overweight young people (15.6%), with Ethiopia providing the lowest prevalence (1.9%) [24]. This variation may be caused by genetic differences, disparities in socioeconomic status, and cultural norms that affect how people perceive their bodies and what they eat. Even though study findings varied, the meta-analysis did not find any statistically significant differences among descriptive studies.

Regarding factors associated with undernutrition, male sex predisposed to both stunting and wasting, occurring at 1.85 and 2.55 times higher than for their female adolescent counterparts, respectively. This might be a consequence of the generally greater growth spurt and energy requirements of male compared to female adolescents [28, 39]. It may also reflect a socio-cultural bias where males are expected to undertake high physical activities to support family subsistence [32]. Similarly, the odds of stunting among adolescents with a history of opportunistic infection was 2.97 times higher than their non-infected counterparts. This finding is consistent with the vicious circle concept of malnutrition and HIV infection, i.e. infection predisposes to malnutrition and malnutrition predisposes to infection [36]. Reasons for this may be that as the disease advances, appetite reduces and disease-related malabsorption problems increase, leading to further vulnerability to opportunistic infection which in turn worsens under-nutrition. Worryingly, only a single trial examined supplementation, showing one year of intervention improved nutritional indices in adolescent with HIV.

Adolescent nutrition has been largely overlooked in intervention and policy research. Most intervention studies have focused on micronutrient supplementation, and few have considered multiple factors in adolescent nutrition [40]. It may indicate that implementation is not progressing as expected. However, there is evidence that nutrition interventions result in beneficial effects [29, 33]. Effective interventions and strategies therefore need to be better implemented to address multiple challenges in communities and sectors. It is supported by a multi-faceted and multi-level policy.

Sustainable nutrition care and support for adolescents with HIV are used to provide adequate nutrient intake to promote normal growth and development during puberty, maintain adequate nutritional status to promote health and prevent disease after physiological growth is complete, promote optimal nutrition and prevention of malnutrition, manage or reduce symptoms of HIV disease, enhance drug compliance and efficacy through diet counseling, prevent food-borne illness, and manage complications associated with HIV and antiretroviral therapy (ART) [41]. Integration of nutrition care and support with the national HIV care and treatment implementation guideline is crucial for effective prevention, building the ART pipeline, ensuring the continuum of care and quality service, and healthy living with HIV.

### Strength and limitation of the review

This assessment has many strengths. An extensive search strategy was implemented and data were analyzed using a rigorous methodology. Explicit inclusion and exclusion criteria related to population and comprehensive outcomes were assessed. Four authors were involved in the quality assessment. As the included studies showed considerable heterogeneity, we performed advanced statistical analysis such as meta-regression to identify possible sources of heterogeneity. Despite the above strengths, this review has some limitations that should be considered before interpreting the results. Since most of the primary studies included in this systematic review and meta-analysis are cross-sectional, no analyses could be undertaken to pool intervention effects as only one single eligible intervention study was found. In addition, the limited number of published studies from low-middle income countries that were retrieved and used for the pooled effect size, may have yielded underrepresentation of data from other low middle-income countries. Other limitations of this study included significant heterogeneity between the primary studies, with review methods limited to those published in the English language. This may have resulted in the exclusion of some essential studies.

## Conclusion and recommendation

In conclusion, the review demonstrates the paucity of research on nutrition in adolescents living with HIV, and the effects of supplementation intervention could not be tested. The review results indicate a high prevalence of stunting and wasting in HIV-infected adolescents, but low proportions of overweight. Opportunistic infection was the sole factor shown by this review to be both significant and amenable to intervention. The inadequate and fragmented nutritional screening and support programs used by government and non-governmental organizations for this population in clinical settings were highlighted.

Review findings have implications for improving the healthy living of adolescents infected with HIV in LMIC through acting on the demonstrated prevalence and identified determinants of undernutrition; by integrating rigorous nutritional screening and assessment modalities into routine services, and developing and evaluating nutritional interventions.

Review findings indicate that comprehensive systems of nutritional assessment and intervention should be integrated within HIV services to be delivered during routine follow-up of adolescents in the ART clinic. Despite its limitations, review findings provide evidence that can be used by policy-makers, health planners and managers, researchers, and planners in LMICs to create an evidence-informed strategy supporting Sustainable Development Goal 1–3.

## Supplementary Information

**Additional file 1: Supplementary Table 1.** Search Strategy “Population, Intervention / Exposure, Comparator / Control, Outcomes (PICO or PEO) Framework”. **Supplementary Table 2.** Characteristics of studies included in this systematic review and meta-analysis, 2022. **Supplementary Table 3.** Distribution of included studies outcomes in this systematic review and metanalysis, 2022. **Supplementary Table 4.** Quality appraisal status of studies included according to JBI characteristics, 2022.

## Data Availability

Most of the data analysed during the systematic review are included in this manuscript.
