# Supplementary material for: Nutritional status and its determinants among adolescents with HIV on anti-retroviral treatment in low- and middle-income countries: a systematic review and meta-analysis
Source: BMC Nutr. 2023 Mar 28;9:60. doi: 10.1186/s40795-023-00714-z (PMC10053752; doi:10.1186/s40795-023-00714-z)
Supplement: Supplementary file 1 — Additional file 1: Supplementary Table 1. Search Strategy “Population, Intervention / Exposure, Comparator / Control, Outcomes (PICO or PEO) Framework”. Supplementary Table 2. Characteristics of studies included in this systematic review and meta-analysis, 2022. Supplementary Table 3. Distribution of included studies outcomes in this systematic review and metanalysis, 2022. Supplementary Table 4. Quality appraisal status of studies included according to JBI characteristics, 2022. [file 40795_2023_714_MOESM1_ESM.docx]

## **Supplementary Tables**

Supplementary Table 1**: Search Strategy “Population, Intervention / Exposure, Comparator / Control, Outcomes (PICO or PEO) Framework”**

| **Populations / Participants (P)** | **Intervention (I) or Exposure (E)** | **Comparators (C) / Controls** | **Outcomes (O)** |
| --- | --- | --- | --- |
| - HIV-positive adolescents on HAART / ART - Living in LMICs | - Any intervention (such as nutrition supplementation, food fortification, nutrition education) or factor (e.g., physical, psychological, social) intended or demonstrated to affect the nutritional status or outcome measures. | - HIV positive on HAART/ ART and HIV negative adolescents who are well-nourished as determined using the WHO/FAO cut-off point | - The magnitude of Nutritional Status (Undernutrition / Malnutrition, Micronutrient deficiency) - Dietary practice |
| **Terms were combined as follows**:  **Line 1**: ("nutritional status" OR “Nutrition*” OR "undernutrition" OR "malnutrition" OR "nutritional deficienc*" OR "energy deficienc*" OR "protein deficienc*" OR "fat deficienc*" OR "micronutrient deficienc*")  AND  **Line 2:** (“adolescent” OR “older children” OR “young adults” OR “teen*” OR “young person” OR “young people”)  AND  **Line 3**: (“HIV” OR “human immunodeficiency virus” OR “Human” OR “Immunodeficiency” OR “AIDS” OR “acquired immunodeficiency syndrome” OR “HAART” OR “highly active antiretroviral therapy” OR “antiretroviral therapy”)  AND   - **Line 4:** (“Angola” OR “Benin” OR “Botswana” OR “Burkina Faso” OR “Burundi” OR “Cameroon” OR “Cape Verde” OR “The central African Republic” OR “Chad” OR “Comoros” OR “Republic of the Congo” OR “Democratic Republic of the Congo” OR “Cote d'Ivoire” OR “Djibouti” OR “Equatorial Guinea” OR “Eritrea” OR Ethiopia” OR “Gabon” OR “The Gambia” OR “Ghana” OR “Guinea” OR “Guinea-Bissau” OR “Kenya” OR “Liberia” OR “Madagascar” OR “Malawi” OR “Mali” OR “Mauritania” OR “Mauritius” OR “Mozambique” OR “Namibia” OR “Niger” OR “Nigeria” OR “Rwanda” OR “Sao Tome and Principe” OR “Senegal” OR “Seychelles” OR “Sierra Leone” OR “Somalia” OR “South Africa” OR “South Sudan” OR “Sudan” OR “Swaziland” OR “Tanzania” OR “Togo” OR “Uganda” OR “Zambia” OR “Zimbabwe”) | | | |
|  | | | |

Supplementary Table 2**: Characteristics of studies included in this systematic review and meta-analysis, 2022**

| **Author and year of publication** | **Country** | **Study setting** | **Aim/Question** | **Sample population** | **Design** | **Data collection methods** | **Nutritional assessments** |
| --- | --- | --- | --- | --- | --- | --- | --- |
|  |  |  |  |  |  |  |  |
| Carlos, et al., 2019 | Brazil | Joana de Gusmao" Children's Hospital (HJG), Florianopolis, Brazil | To verify the association between anthropometric indicators and fat percentage estimated by using different reference techniques (DXA and ADP) in children and adolescents diagnosed with HIV, and by controlling factors related to treatment, maturation, and physical activity. | n=62, age range 8 - 15, mean 12.8 years. 28 male, 34 females | Quantitative correlational study | Anthropometric measurements: • Skinfolds using skinfold callipers skinfolds calliper (Cescorf, Porto Alegre, Brazil) • Body parameters measured were using an inelastic tape  • Body weight was measured using a portable digital Electrical Bathroom scale  • Height measured using a stadiometer  • All anthropometric indicators were measured based on recommendations of the International Society for the Advancement of Kin-anthropometry (ISAK) by a single level-1 ISAK anthropometrist.  • Body fat was measured using dual X-ray absorptiometry (DXA), air displacement plethysmography (ADP), and computed tomography. | Skinfolds (abdominal, triceps, subscapular, calf), perimeter relaxed arm (PRA), waist circumference (WC), perimeter neck, body mass index (BMI), waist-to-height ratio, conicity index, and body adiposity index (BAI) |
| Bissigo, et al., 2016 | Brazil | HIV/AIDS Pediatric Ambulatory of the Hospital de Clinicas de Porto Alegre | To evaluate macro and micronutrient intake of adolescents living with HIV/AIDS in use of antiretroviral therapy and compare it to the Dietary Reference Intakes. | n=39, mean age - 15 years, 20 male, 19 females | Cross-sectional study | • 24-hour dietary recall standardized structured interview questionnaires were used to assess all food and drink consumed in the previous 24 hours to determine the nutrient intake  • medical record reviews about clinical status by using a standardized format | 24-hour Recall Dietary Intake data compared to Dietary Reference Intakes (DRI) (2005). Total fat, saturated fat, and cholesterol intakes were evaluated compared to AHA recommendations (Lichtenstein AH, et al., 2006). |
| Castro, et al., 2018 | Brazil | Brazilian regional referral hospital for HIV treatment. | To determine the validity of body composition analysis by BIA compared to dual-energy X-ray absorptiometry (DXA) and air displacement plethysmography (ADP) in HIV-infected children and adolescents | n= 64, mean age - 12.22 years, 29 males, 35 females | Cross-sectional validity study | • Body composition assessments using DXA performed with GE Lunar Prodigy Advance equipment and ENCORE 2004, version 8.10.027 (GE Lunar Corporation, Madison, WI, USA)  • Body mass measured with a digital scale integrated with the Bioelectrical Impedance Analysis (BIA) equipment • BIA procedures performed with In Body-720 octapolar multifrequency equipment (Biospace, Los Angeles, CA, USA)  • Stature measured using an AlturaExata stadiometer (Belo Horizonte, Brazil) • From these procedures, body mass index (BMI) was calculated  • Air displacement plethysmography (ADP) procedures performed with BOD-POD equipment (Life Measurement Inc., Concord, CA, USA) | Body Mass Index, Total Body Composition [FM- BAI (Kg), FM ADP(kg), FM DXA(Kg), %BF ADP, FFM BIA(kg), FFM ADP(kg), Lean Soft Tissue Mass (LSTM) BIA(kg), Bone Mineral Content(BMC) BIA(kg), BMC DXA(kg)] |
| Darshit, et al., 2020 | Uganda | Pediatrics Infectious Diseases Clinic | To assess the nutrition status and associated factors among HIV-positive adolescents on an Atazanavir-based regimen attending an urban clinic in Uganda | n=132, mean age - 17 years, 86 males, 46 females | Cross-sectional study | • A structured pretested interviewer-administered questionnaire in English and Luganda languages was used to collect sociodemographic data.  • Anthropometric Assessment:   1. Weight was measured using an electronic Seca scale with daily calibration and recorded to the nearest 0.1 kg.   2. Height measurements were taken at maximum inspiration using a standardized erect stadiometer recorded to the nearest 0.1 cm. REDCap ® platform was used for data management. | BMI-for-Age (BAZ) and Height-for-age (HAZ) indices and analyzed using WHO Anthro Plus software. |
| David, et al., 2020 | Cameroon | Mother and Child care Centre of the Chantal BIYA Foundation (i.e. Specialized unit for the care of HIV-infected children and mothers) | To describe and compare the growth of children living with HIV with those not infected and identify the associated factors | n=76, aged range - 10-19 years, 35 male, 41 female, 19 HIV-Infected cases, and 57 uninfected controls matched by sex and age plus or minus 02 months. | Case-control study | This study used three methods of assessment by using standardized tools: 1) Clinical evaluations/assessment on past history, clinical and biological stages of the disease; 2) Anthropometric parameters assessment based on WHO Anthro software requirements. 3) Individual Dietary Diversity assessment with food group intake | Height-for-Age, Weigh-for-Height, Weight-for-Age measurements indices, and Individual Dietary Diversity Score (IDDS) to determine the dietary diversity and nutrient intake |
| Dos Reis, et al., 2015 | Brazil | HIV/AIDS out-patient clinic of the Institute of Child Health, São Paulo. | To investigate the relationship between anthropometric parameters and body composition of perinatally HIV-infected children and adolescents under HAART, according to use and non-use of protease inhibitors. | n=89, aged range- 6 - 19 years, 43 male, 46 females | Cross-sectional study | • Demographic, socioeconomic, clinical, and anthropometric data were collected by using a structured questionnaire, Physical examination, and Medical records review. • The anthropometric data were taken by this article:  • Weight measured using a portable electronic scale   • Height measured with a Leicester stadiometer   • Mid-arm, waist, and neck circumferences were obtained with an inextensible centimeter-graded measuring tape   • Triceps skinfold thickness was taken with a calibrated Lange® adipometer (Beta-Technology, Santa Cruz, CA, USA)  • The mid-arm fat and muscle areas were calculated using the equations recommended by Frisancho(1981).   • Body fat percentage obtained by bioelectrical impedance analysis (BIA) using the Biodynamics® analyser (model 310, Seattle, WA, USA). | BMI for Age (z score), Height-for-age (z score), Waist circumference for age (percentile), Neck circumference for age (percentile), Triceps skinfold thickness (percentile), body fat percentage, upper-arm fat area, and upper-arm muscle area |
| Hillesheim., et al., 2014 | Brazil | Hospital Infantil Joana de Gusma˜o | To investigate the nutritional status and dietary intake of HIV-infected children and adolescents and the relationship between nutritional status and dietary intake and CD4+ T-cell count and viral load. | n=49, mean age - 12.6 years, 25 male, 249 females | Observational study | • Face-to-face interviews using a standardized questionnaire for socio-demographic data • Medical record review to obtain Immunological data • Anthropometric Measurement- Weight using a calibrated digital scale • Dietary intake was collected using the Food Frequency Questionnaire for Adolescents (FFQA) validated (Slater, et al, 2003). | • Anthropometry indices: height-for-age (H/A) z-score and body mass index-for-age (BMI/A) • Total energy intake (TEI), and nutrient intake from FFQ Dietary intake assessment using the software Programa de Apoio a` Nutric¸a˜o – NutWin 1.6.0.7 (Federal University of Sa˜o Paulo, Brazil). |
| Jesson, et al., 2015 | Central and West-African | HIV-care programs supported by the Sidaction Growing Up Programme | To assess the prevalence and associated factors of acute and chronic malnutrition among HIV-infected children | n= 684, age range - 10 - 19 years, 349 male, 335 females | Cross-sectional study | • Medical records review was used to collect socio-demographic characteristics, Anthropometric, clinical data, and nutritional support (flour, powdered milk, solid or semi-solid foods, or Ready-to-Use Therapeutic Food [RUTF]) | Anthropometry indices: Weight-for-Height Zscore(WHZ) or BMI-for-Age Z-score (BAZ), Height-for-Age Z-score (HAZ) and analyzed using WHO Anthro Software (version 3.2.2) and WHO AnthroPlus |
| Lwanga, et al., 2015 | Uganda | The AIDS Support Organization (TASO) HIV care services in HIV-care Programs in Urban and Rural Uganda | To assess the nutritional status of HIV-infected adolescents receiving HIV care services at six The AIDS Support Organization (TASO) in Uganda. | n=200, age range- 10-19 years, 77 male, 123 females | Cross-sectional study | • Structured interviewer-administered questionnaire and anthropometric measurements: Weight using Seca scales and Height using height board | Anthropometry indices: BMI-for-Age (BAZ) and Height-for-age (HAZ) |
| Murray, et al., 2020 | Myanmar | Outpatient HIV testing and care in a rural community in south-eastern Myanmar | To examine the patient’s high levels of viral repression, malnutrition, and second-line ART use in adolescents living with HIV | n= 177, age range - 10 -19 years, 81 males and 96 females for quantitative; and 12 adolescents and 10 caregivers’ sub-sample for the qualitative study | Mixed Method Study | • The quantitative component, includes clinical history, medical examination, and laboratory investigation conducted by a medical doctor and counsellor.  • In-depth interviews (IDIs) and two focus group discussions (FGD) for collecting qualitative data.  • For clinical data: a physical and neurological exam was conducted for each patient, and a laboratory investigation was conducted on whole blood samples.  • A Patient Health Questionnaire A (PHQ-A), a modified version of the PHQ-9, a standardized assessment tool, was used as a depression screening tool by a trained HIV counsellor  • Lipodystrophy is based on a physician’s diagnosis of abnormal fat distribution (lipoatrophy or lipo-hypertrophy). | **Anthropometric indices:** Body mass index (BMI) scores, and Lipodystrophy physical diagnosis abnormal fat distribution |
| Niasse F., et al., 2020 | Senegal | Study of Nutrition and Activity in Childcare (SNAC) Settings | To assess the overall acceptability and perception of a Ready-to-use food (RUF)-based therapy and risk factors associated with sub-optimal RUF intake in HIV-infected undernourished adolescents in Senegal | n=173, aged range - 5 - 18 years (median age: 12.5 years) who are under active follow-up and presenting with acute malnutrition, i.e., 104 MAM and 69 SAM | Clinical Trial Study | • Medical Record Review: Clinical and therapeutic characteristics  • The Household food insecurity access scale (HFIAS) was used to assess food insecurity.  • 24-h recall of Ready-to-use food (RUF) intake at enrolment  • At week 2, a structured questionnaire covering 5 topics, organoleptic appreciation of RUF, mode of intake, 24-h recall of RUF intake, self-stigma associated with RUF intake, and RUF sharing, was administered primarily to participants ≥7 years or caregivers below that age.  • Questions about RUF sharing were administered separately to participants ≥7 years and caregivers. | **Anthropometry indices:** Weight, Height, BMI Z-score |
| Rao & Ragireddy, 2020 | India | ART centre | To assess the nutritional status and nutritional supplementation in children with HIV at ART centre | n=100, Mean age (SD)- 11.02 years, 44 males, 56 females | Observational study | Clinical and anthropometric data (as baseline) and after supplementation were taken | **Anthropometry indices:** Height-for-age (HFA), Weight-for-age (WFA), BMI for-age and computed using standard references [using WHO Anthro software (version 3.2.2.) and WHO Anthro Plus software] |
| Ramalho LC., et al., 2014 | Brazil | Paediatric Immunodeficiency Clinic at the State University of Campinas Hospital, Campinas, Sa˜o Paulo, Brazil, a tertiary referral centre | To compare body composition outcomes between HIV-infected children and adolescents on ART and healthy controls, and to evaluate their association with clinical, immunological, and lifestyle variables, within the HIV-infected group | n= 94, 55 males aged range-7.68–19.76 years (median 12.72), and 39 females aged 8.15–18.35 years (median 12.07), and 364 healthy children and adolescents (178 males and 186females) comprised the control group | Cross-sectional study | • Anthropometric evaluations and blood sample collections after a 12-hour fast • Height and weight were measured according to recommendations from the Anthropometric Standardization Reference Manual (Lohman TG, et al., 1988).  • BMI international standards are used to determine overweight, obesity, and thinness (Cole et al. 2007).  • Waist and hip circumferences with flexible metric tape (precision of 1 mm).  • Skinfold measurements were performed with a Lange calliper (Beta Technology Inc, Cambridge, MA, USA), according to procedures established by Lohman (Lohman et al. 1988), and skinfold thickness equations were calculated for fat percentage.  • Lipodystrophy is defined by clinical examination Clinical and immunological HIV categories are defined according to CDC standards, adapted by the Brazilian Ministry of Health.  • Physical activity was measured with the physical activity questionnaire for children, 20 for patients up to 12 years old, and the international physical activity questionnaire, 21 for patients older than 12 years.  • Food energy intake is measured by 24-hour recall and its adequacy was evaluated according to Recommended Dietary Allowances | **Anthropometry:** Weight, height, body mass index (BMI), waist and hip circumferences, Skinfold thickness, Fat percentage, BMD and body composition (percent body fat and lean mass), 24-hour recall, and energy and nutrient intake. |
| Schtscherbyna, et al., 2012 | Brazil | Brazilian cohort of vertically HIV-infected adolescents followed-up at the Infectious Diseases Service of the HUCFF-UFRJ | To assess the prevalence and factors associated with low bone mineral density (BMD) in HIV-infected adolescents | n=74, mean age - 17.3 years, 33 males, 41 females | Cross-sectional study | • DXA scans were performed on the lumbar spine (LS) and total body (TB) to assess Bone Mineral Density (BMD) and body composition (percent body fat and lean mass), using Prodigy software v. 11.40, adequate for child and adolescent assessment.  • single 24-h recall dietary intake conducted by a trained registered nutritionist, using measuring cups, spoons, and portion-size images to increase the accuracy of the recall. • The household measurements are converted into grams and millilitres for quantitative analysis of the energy and nutrient intake using the software Avanutri online (version online; 2010, Rio de Janeiro, Brazil).  • Only energy, macronutrients (carbohydrate, protein, and lipid), calcium, and vitamin D results are presented here. Calcium and vitamin D consumption was categorized according to the estimated average requirement (EAR) | **Anthropometry:** Weight, height, body mass index (BMI), Bone Mineral Density and body composition (percent body fat and lean mass), 24-hour recall, and energy and nutrient intake |
| Sewale, et al., 2018 | Ethiopia | HIV-care service centre of three public hospitals in East and West Gojjam Zones of North west Amhara | To assess the magnitude of malnutrition and associated factors among HIV infected children in Amhara Regional State, and Northwest Ethiopia | n=372, mean age -10 years, 186 male, 186 females | Comparative Cross-sectional study | Record review and face-to-face interviews by using the structured adapted tool. Anthropometric measurements: Weight and height measured by using standardized tools and calibration | **Anthropometry**: weight-for-height Z-score, weight-for-Age Z-score, Height-for-Age Z-score, Body Mass-for-Age Z-score and computed by using WHO anthro and WHO anthroplus software |
| Shiferaw, et al., 2020 | Ethiopia | Two public hospitals and three health centres who provide ART services | To assess the prevalence and predictors of stunting and thinness among adolescents receiving anti-retroviral therapy (ART) in Hawassa city, Southern Ethiopia | n=260, age range 10–19 years, 127 males, 133 females | Cross-sectional study | • Data was collected using an interviewer-administered pretested questionnaire by trained personnel, directly from the primary caregivers and the adolescents while ART follow-ups.  • Dietary diversity using a standard tool of the Food and Agriculture Organization of the United Nations (FAO) as the number of food groups consumed over the preceding day, out of the standard list of 12 groups.  • Household food insecurity was measured and classified into four ordinal categories (secure, mild, moderate, and severe insecurity) using the standard Household Food Insecurity Access Scale (HFIAS)  • Body height and weight are measured via calibrated tools following standardized procedures.  • Weight was measured by a digital scale to the nearest 0.1 kg and height was measured using a portable stadiometer to the nearest 0.1 cm.  • Clinical characteristics (CD4 count, HIV staging, occurrence of opportunistic infections) extracted from individual medical records | **Anthropometry indices**: Height, Weight, BMI-for-age index, HFA z-score, BMI-for-age z-score |
| Yasuoka, et al., 2020 | Cambodia | National Pediatric Hospital, which provides pediatric ART in Phnom Penh | To examine nutritional status of school-age children living with HIV in Phnom Penh, Cambodia, and identify factors associated with their nutritional status, especially taking their dietary diversity | n=298, age range 6–15 years, 152 males, 146 female | Cross-sectional study | • Open Data Kit 2.0 was used to directly record study participants’ responses to questionnaires (available at https://opendatakit.org/use/2_0_tools/).  • Body weight, height, and clinical data were recorded on paper-based forms and entered electronically by data management assistants at the National Pediatric Hospital.  • Dietary diversity using the standard tool and a dietary diversity score (DDS) calculated by summing the number of food groups consumed by each child over the previous 24-h recall period.  • Overall health-related quality of life was measured using pediatric Quality of Life Inventory 4.0 (PedsQL™ 4.0), which has been validated for children living with HIV | **Anthropometry indices:** Height, Weight, BMI-for-age index, HFA z-score, BMI-for-age z-score, 24-hour dietary recall |

Supplementary Table 3**: Distribution of included studies outcomes in this systematic review and metanalysis, 2022**

| **Author and year of publication** | **Nutritional Status (Prevalence Rate)** | **Factor associated with the existed nutritional status** | **Nutritional Intervention** | **Effects of Nutritional Intervention** |
| --- | --- | --- | --- | --- |
|  |  |  |  |  |
| Carlos, et al., 2019 |  |  |  |  |
| Bissigo, et al., 2016 |  |  |  |  |
| Castro, et al., 2017 |  |  |  |  |
| Darshit, et al., 2020 | • Stunting- 31(23.7%),  • Wasting - 10(7.6%),  • Overweight - 4(3.8%) | • Availability of parent (AOR= 3.70, 95% CI: 1.20-11.37, pv=0.023) • Level of Educational status (AOR: 0.40, 95%CI: 0.17-0.95, Pv=0.037) |  |  |
| David, et al., 2020 | • Stunting- 15(36.6%),  • Underweight - 4(18.2%),  • Overweight - 3(7.3%) | • Biological Disease grade 3 (OR = 5.67, P = 0.34),  • Low nutritional intake (OR = 7.8, P = 0.12),  • Gastroenteritis (OR = 3.5, P = 0.29),  • Elevated viral load (OR = 5, P = 0.2) |  |  |
| Dos Reis, et al., 2015 | • Stunting- 24(20.9%),  • Wasting - 4(3.5%),  • Overweight - 18(15.6%) |  |  |  |
| Hillesheim., et al., 2014 | • Stunting- 3(6.1%),  • Wasting - 1(2.0%),  • Overweight - 3(6.1%) | • Energy intake - 50.8% above the estimated energy requirement  • Inadequate intake of polyunsaturated fat, cholesterol, fiber, calcium, and vitamin C was present in 100%, 57.1%, 40.8%, 61.2%, and 26.5% of the sample, respectively.  • MLR analyses revealed that energy intake was correlated with CD4+ T-cell count (r- 0.33; p-0.028) and viral load (r - 0.35; p -0.019). |  |  |
| Jesson, et al., 2015 | • Stunting - 163(23.8%),  • Wasting- 69(10.0%) | **Factor associated with acute malnutrition (wasting):** • Male gender [AOR=2.27, 95%CI (1.52, 3.41)],  • Severe immunodeficiency [AOR=2.07, 95%CI (1.25, 3.42)],  • Absence of ART [AOR=1.70, 95%CI (1.01, 2.84)]; **Factors of chronic malnutrition (Stunting):** • Male gender [AOR=1.56, 95%CI (1.20, 2.03)]  **Factor associated with mixed malnutrition (Underweight):** • Male gender [AOR = 2.60, 95%CI (1.64, 4.10)],  • Severe immunodeficiency [AOR = 2.43, 95%CI (1.40, 4.23)]  • Recent ART initiation (<6 months) [AOR = 2.54, 95%CI (1.17, 5.55)]. |  |  |
| Lwanga, et al., 2015 | • Stunting - 72(36.2%),  • Wasting- 36(18.0%) | The risk factors for stunting  • Male Gender (AOR: 4.0; 95% CI: 1.81- 7.02) and • Living in rural settings (AOR: 6.0; 95% CI 2.70-12.16) |  |  |
| Murray, et al., 2020 | • Severe Underweight (BMI < 16) - 104 (59%), • Moderate underweight - 22(12%), • Mild underweight - 18(10%) |  |  |  |
| Niasse F., et al., 2020 |  |  | **Medication/ Supplement:** RUF, Plumpy Nut® and Plumpy Sup®. **Provision:** 2 weeks RUF Ration based on age **Follow-up:**  every 2 weeks for clinical assessment and 9 months in decentralized clinics or 12 months in Dakar until **recovered** or **discontinued**. Median follow-up duration was 66 days (21–224). **Monitoring:** of adherence to RUF over the preceding 2 weeks and outcome |  |
| Rao & Ragireddy, 2020 |  |  | Nutrition supplementation: ** 2 scoops of Protein powder/day** • Total amount: 60gms • Total calorie: 210 kcal • Total protein: 19.2gms ** Peanut chikki – one serving**  • Total amount:30gms • Total calorie: 150 kcal • Total protein: 4gms ** Total Calorie: 360Kcal ** Total proteins: 23.2gm | After giving the nutritional supplementation for a period of **one year**: •statistically significant improvement in height-for-age, weight-for-age, and the Z scores of height-for-age, weight-for-age, and BMI-for-age, but not Mean Value of BMI-for-Age |
| Ramalho LC., et al., 2014 | • Stunting- 24(25.5%),  • Wasting - 21(22.3%),  • Overweight - 6(6.4%) | • When compared with subjects in the control group, HIV-infected patients had:  => higher risk of short stature /stunting [OR=5.33, 95%CI (2.83, 10.04)]  => thinness /malnutrition [OR=4.7, 95%CI (2.44. 9.06)],  => higher waist-to-hip ratios (medians 0.89 versus 0.82 for boys and 0.90 versus 0.77 for girls, P, 0.001), and  => lower prevalence of overweight or obesity [OR= 0.33, 95%CI (0.14, 0.78)]  • Patients in CDC clinical category C had a higher risk of short stature (OR = 3.68; 95% CI 1.39–9.73).  • Children on Protease Inhibitors had a higher risk of lipoatrophy and malnutrition (OR =3.5; 95% CI 1.37–8.95 and OR =3.51; 95% CI 1.07–11.44, respectively).  • Abdominal lipohypertrophy significantly increased in children in immunological category 3 (OR=2.5; 95% CI 1.06–5.91)  • Independently of gender, children in immunological category 3 had higher waist-to-hip ratios (medians 0.91 versus 0.89, P=0.004).  • Older age was associated with higher waist circumference (P = 0.58, P, 0.001), higher SS/TR skinfold ratios (P =0.32, P = 0.021), and lower waist-to-hip ratios (P = 20.24, P = 0.003). |  |  |
| Schtscherbyna, et al., 2012 |  | • Body composition and nutritional status were positively associated with BMD Z-scores, especially in females.  • Lumbar spine (LS) and total body (TB) BMD Z-scores were positively correlated with weight, body mass index (BMI), BMI Z-score, total body fat, and nutritional status.  • Patients on tenofovir had lower lumbar spine (LS) and total body (TB) BMD Z-scores.  • Time on tenofovir was indirectly correlated with LS and TB BMD Z-scores.  • No difference was found regarding levels of calcium, parathyroid hormone, or 25-hydroxyvitamin D according to BMD status |  |  |
| Sewale, et al., 2018 | • Wasting - 224(60.2%) | Significant factors of Malnutrition: • Having good individual dietary diversity (AOR = 0.474, 95% CI (0.26, 0.86)).  • Sex of child (AOR = 2.37, 95% CI (1.34, 4.20), • Age 10 - 15 years (AOR=0.05, 95% CI (0.01, 0.25),  • Comorbidity disease (AOR = 0.34, 95% CI (0.15, 0.800)),  • Oral ulcer (AOR = 2.30, 95% CI (1.41, 7.60)),  • Diarrhea (AOR = 3.30. 95%CI (1.41, 7.72)) and • History of hospital admission (AOR = 0.34, 95% CI (0.13, 0.84)) |  |  |
| Shiferaw, et al., 2020 | • Stunting- 86(33.1%),  • Wasting - 52(20.0%),  • Overweight - 5(1.9%) | • History of recent opportunistic infection was the only significant predictor of thinness (AOR=3.21; 95% CI: 1.54, 6.73) |  |  |
| Yasuoka, et al., 2020 | • Stunting- 139(46.6%),  • Wasting - 39(13.1%) | Factors associated with children’s stunting were: • Age (AOR= 2.166, 95% CI: 1.151, 4.077),  • Household wealth (AOR= 0.543, 95%CI: 0.299, 0.986),  • Duration of receiving ART (AOR 0.510, 95%CI: 0.267, 0.974), and  • Having disease symptoms during the past 1 year (AOR 1.871, 95%CI: 1.005, 3.480)  Factor associated with wasting was: • Male (AOR 5.304, 95%CI: 2.210, 12.728) |  |  |

Supplementary Table 4**: Quality appraisal status of studies included according to JBI characteristics, 2022**

| **Author, year** | **Study design** | **Q1** | **Q2** | **Q3** | **Q4** | **Q5** | **Q6** | **Q7** | **Q8** | **Q9** | **Q10** | **Q11** | **Q12** | **Q13** | **Score per tool Item** |
| --- | --- | --- | --- | --- | --- | --- | --- | --- | --- | --- | --- | --- | --- | --- | --- |
| Carlos A.S., et al, 2019 | Analytical, quantitative, correlational study | 1 | 0 | 0 | 1 | 0 | 1 | 1 | 1 | - | - | - | - | - | 5 |
| Fernanda Bissigo P., et al., 2016 | Cross-sectional study | 1 | 1 | 1 | 1 | 0 | 0 | 1 | 1 | 1 | - | - | - | - | 7 |
| Castro J. A. C., et al., 2017 | Cross-sectional validity study | 1 | 1 | 0 | 0 | 1 | 0 | 1 | 0 | 1 | - | - | - | - | 5 |
| Darshit D., et al., 2020 | Cross-sectional study | 1 | 1 | 0 | 1 | 1 | 1 | 0 | 0 | 1 | - | - | - | - | 6 |
| David Chelo, et al., 2020 | Case-control study | 1 | 1 | 1 | 0 | 0 | 1 | 1 | 0 | 1 | 1 | - | - | - | 7 |
| Dos Reis, LC., et al., 2015 | Cross-sectional study | 1 | 1 | 1 | 1 | 1 | 1 | 0 | 1 | 1 | - | - | - | - | 8 |
| Hillesheim, E., et al, 2014 | Observational study | 1 | 1 | 0 | 1 | 1 | 0 | 1 | 1 | 1 | - | - | - | - | 7 |
| Jesson et al., 2015 | Cross-sectional study | 1 | 1 | 0 | 1 | 1 | 0 | 0 | 1 | 1 | - | - | - | - | 6 |
| Lwanga F., 2015 | Cross-sectional study | 1 | 1 | 0 | 1 | 1 | 1 | 1 | 1 | 1 | - | - | - | - | 8 |
| Murray J., et al., 2018 | Mixed Method Study | 1 | 1 | 1 | 1 | 1 | 0 | 1 | 0 | 1 | - | - | - | - | 7 |
| Niasse F., et al., 2020 | Clinical Trial Study | 1 | 1 | 1 | 1 | 0 | 1 | 1 | 0 | 1 | 1 | 0 | 1 | 1 | 10 |
| Rao Muddana N. and Ragireddy A., 2020 | Observational study | 1 | 1 | 0 | 0 | 1 | 0 | 1 | 0 | 1 |  |  |  |  | 5 |
| Ramalho LC., et al., 2014 | Cross-sectional study | 1 | 1 | 0 | 1 | 1 | 1 | 1 | 1 | 1 | - | - | - | - | 8 |
| Schtscherbyna A., et al., 2012 | Cross-sectional study | 1 | 1 | 1 | 0 | 1 | 1 | 0 | 1 | 1 | - | - | - | - | 7 |
| Sewale Y., et al., 2018 | Cross-sectional study | 1 | 1 | 0 | 0 | 1 | 0 | 1 | 1 | 1 | - | - | - | - | 6 |
| Shiferaw H., and Gebremedhin S., 2020 | Cross-sectional study | 1 | 1 | 0 | 1 | 1 | 0 | 1 | 1 | 1 | - | - | - | - | 7 |
| Yasuoka J., et al., 2020 | Cross-sectional study | 1 | 1 | 1 | 1 | 0 | 0 | 1 | 0 | 1 | - | - | - | - | 6 |
| NB.- JBI critical appraisals tools for all studies are used as shown in the link below <https://jbi.global/critical-appraisal-tools> | | | | | | | | | | | | | | | |
